# Characterization of the mitochondrial genome of *Favonigobius reichei* (Perciformes, Gobiidae)

**DOI:** 10.1080/23802359.2020.1791014

**Published:** 2020-07-20

**Authors:** Xiongbo He, Murong Yi, Yunrong Yan, Yu-Min Ju

**Affiliations:** a College of Fisheries, Guangdong Ocean University, Zhanjiang, China; b Marine Resources Big Data Center of South China Sea, Southern Marine Science and Engineering Guangdong Laboratory (Zhanjiang), Zhanjiang, China; c Guangdong Provincial Engineering and Technology Research Center of Far Sea Fisheries Management and Fishing of South China Sea, Guangdong Ocean University, Zhanjiang, China; d Center of Marine Fisheries Information Technology, Shenzhen Institute of Guangdong Ocean University, Shenzhen, China; e National Museum of Marine Biology and Aquarium, Checheng Township, Taiwan; f Graduate Institute of Marine Biology, National Dong Hwa University, Shoufeng Township, Taiwan

**Keywords:** *Favonigobius reichei*, mitochondrial genome, next-generation sequencing

## Abstract

*Favonigobius reichei*, belongs to the family Gobiidae, which is widely distributed in estuarine and marine waters of the coasts of the Indian and the western Pacific Oceans. The entire mitochondrial genome of *F. reichei* is 16,415 base pairs (bp) in length and contained 13 protein-coding genes (PCGs), 2 ribosomal RNA (rRNA) genes, and 22 transfer RNA (tRNA) genes. The overall base composition is 27.71% A, 26.78% T, 28.02% C, and 17.49% G, showing AT-rich feature (54.49%). Phylogenetic analysis based on 13 PCGs shows the *F. reichei* has the closest evolutionary relationship with *Myersina macrostoma*. This work provides a valuable mitogenome resource for better understanding of the conservation genetics, environmental DNA, and population studies in the family Gobiidae.

The family Gobiidae, also known as one of the largest vertebrate families, with 1916 valid species in five subfamilies exists according to Fricke et al. (2019). *Favonigobius reichei* (Bleeker 1854), the Indo-Pacific tropical sand goby, is widely distributed in estuarine and marine waters of the coasts of the Indian and the western Pacific Oceans (Froese and Pauly 2019). The genus *Favonigobius* currently comprises nine recognized species, while only one *Favonigobius* mitogenome (*F. gymnauchen*) has been completely sequenced; denser species sampling has been demonstrated to facilitate for species phylogenetic analyses and evolutionary analyses. Here, we assembled the complete mitochondrial genome of *F. reichei* for phylogenetic and evolutionary investigations of *Favonigobius* and Gobiidae.

Specimens of *F. reichei* were collected from the LingShui city in Hainan Island (18.37°N, 109.91°E) and were vouchered at College of Fisheries, Guangdong Ocean University with number GOU103018. The total genomic DNA was extracted from muscle tissue using a TIANamp Marine Animals DNA Kit (Tiangen Biotech, Beijing, China) and next-generation sequencing was described in previous publication (Chiu et al. 2018; Yi et al. 2019). Totally, it produced 24,208,368 clean reads with a size of 3,631,255,200 base pairs (bp) by Illumina HiSeq platform (Illumina, San Diego, CA). Protein-coding genes, ribosomal RNA (rRNA) genes, and transfer RNA (tRNA) genes were identified using MITOS (Bernt et al. 2013) tool and manually inspected.

The complete mitochondrial genome of *F. reichei* is 16,415 bp in length, containing 26.78% of T, 28.02% of C, 27.71% of A, and 17.49% of G (GenBank accession number: MN617828). It contains the typical set of 13 protein-coding genes (PCGs), 22 tRNAs, 2 rRNAs (12S and 16S rRNAs) and a D-loop control region. All PCGs start with ATG, except for COI, which used GTG as the start codon. The stop codon of seven protein coding genes (COI, ATP8, ATP6, ND4L, and ND5) is TAA, whereas the ND1, ND2, ND3, and ND6 genes use TAG as the stop codon. The remaining PCGs (COII, COIII, ND4, and Cyt *b*) have incomplete stop codons T (Figure 1). The 22 tRNA genes have lengths ranging from 68 (tRNA^Phe^, tRNA^Ser^) to 75 (tRNA^Lys^) bp and can fold into a typical cloverleaf structure except tRNA^Ser^ (AGY), which lacks a dihydrouridine arm.

**Figure 1.**
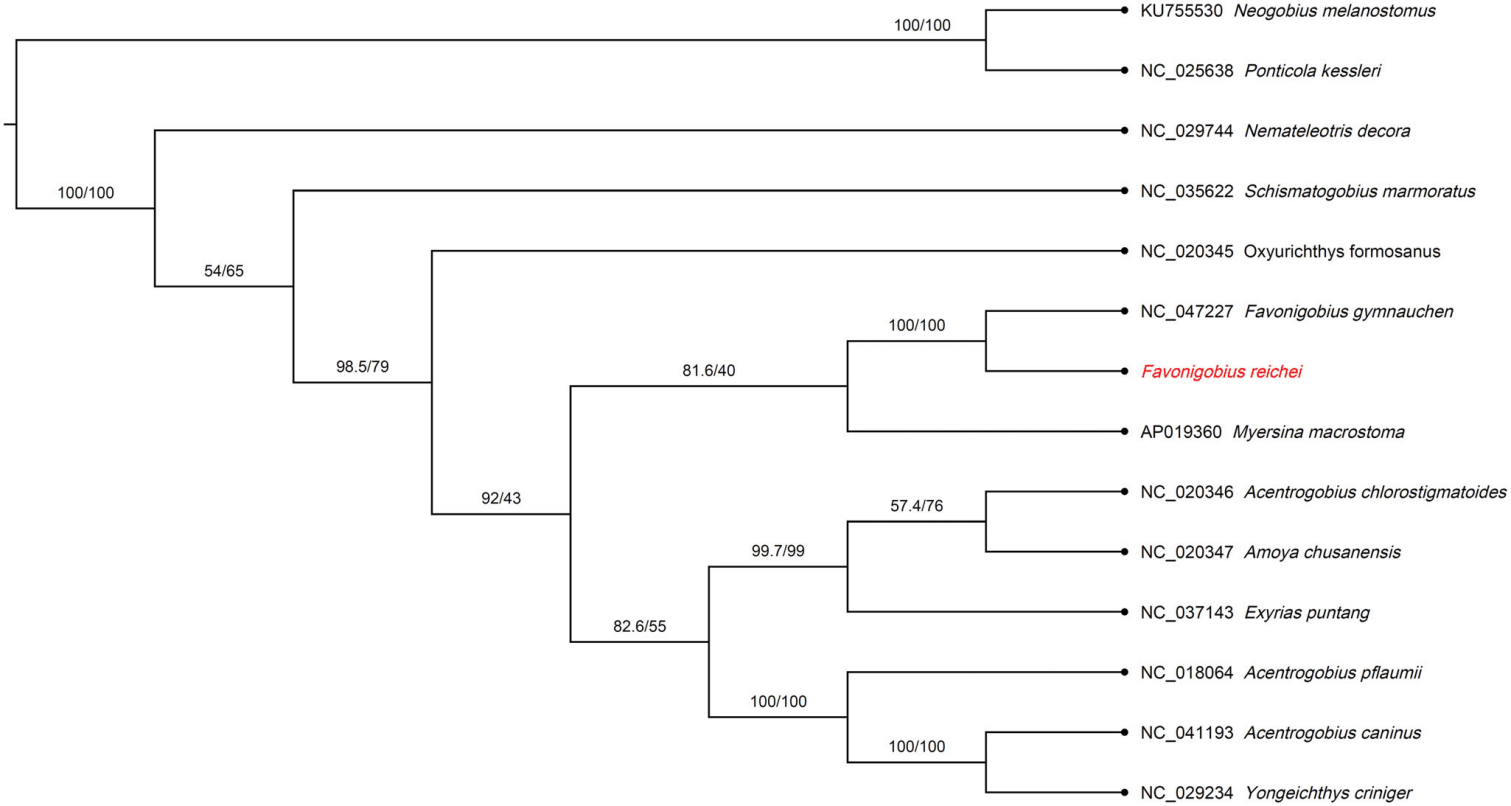
Phylogenetic tree of *Favonigobius reichei* and 13 related species in Gobiidae based on the 13 concatenated nucleotide sequences of the entire protein-coding genes (PCGs) by maximum likelihood (ML) analysis. The numbers on the nodes are the bootstrap values/SH-like aLRT analyses. GenBank accession numbers are given before the species name.

To determine the phylogenetic position of *H. melapterus*, phylogenetic tree was reconstructed by maximum likelihood (ML) methods using PhyloSuite (Zhang et al. 2020). Phylogenetic relationships based on the concatenated set of 13 PCGs sequences of the 13 Gobiidae species indicates that *F. reichei* has the closest evolutionary relationship with *F. gymnauchen* and *Myersina macrostoma* (Figure 1). This study provides a valuable mitogenome resource for better understanding of the conservation genetics, environmental DNA, and population studies in the family Gobiidae.

## Data Availability

The data that support the findings of this study are openly available in GenBank of NCBI at https://www.ncbi.nlm.nih.gov, reference number [Accession number: MN617828].

## References

[CIT0001] Bernt M, Donath A, Juhling F, Externbrink F, Florentz C, Fritzsch G, Putz J, Middendorf M, Stadler PF. 2013. MITOS: improved de novo metazoan mitochondrial genome annotation. Mol Phylogenet Evol. 69(2):313–319. 22982435 10.1016/j.ympev.2012.08.023

[CIT0002] Chiu YW, Chang CW, Shen KN, Ju YM, Lin HD. 2018. Complete mitochondrial genome and the phylogenetic position of the pelagic octopus *Tremoctopus violaceus* (Mollusca: Tremoctopodidae). Mitochondrial DNA Part B. 3(2):1248–1249. 33474480 10.1080/23802359.2018.1532347 PMC7799641

[CIT0003] Fricke R, Eschmeyer WN, Van der Laan R, editors. 2019. Eschmeyer’s catalog of fishes: genera, species, references; [accessed 2020 Mar 10]. http://researcharchive.calacademy.org/research/ichthyology/catalog/fishcatmain.asp.

[CIT0004] Froese R, Pauly D, editors. 2019. FishBase, version (12/2019). http://www.fishbase.org.

[CIT0005] Yi M, Gu S, Luo Z, Lin HD, Yan Y. 2019. Characterization of the complete mitochondrial genome of the coral reef fish, *Hemigymnus melapterus* (Pisces: Labridae) and its phylogenetic implications. Mitochondrial DNA Part B. 4(2):4168–4169. 33366366 10.1080/23802359.2019.1693302 PMC7707650

[CIT0006] Zhang D, Gao F, Li WX, Jakovlić I, Zou H, Zhang J, Wang GT. 2020. PhyloSuite: an integrated and scalable desktop platform for streamlined molecular sequence data management and evolutionary phylogenetics studies. Mol Ecol Resour. 20(1):348–355. 31599058 10.1111/1755-0998.13096

